# Shadow AI in Swedish Health Care: Qualitative Analysis of Physicians’ Free-Text Answers

**DOI:** 10.2196/93484

**Published:** 2026-07-28

**Authors:** Lena Petersson, Luís Irgang, Ingela Mauritzon, Magnus Holmén

**Affiliations:** 1 The Department of Health and Sport, School of Health and Welfare, Halmstad University, Box 823, Halmstad, Halland, 301 18, Sweden, 46 702055024; 2 School of Business, Innovation, and Sustainability, Halmstad University, Halmstad, Halland, Sweden

**Keywords:** artificial intelligence, health care, ChatGPT, generative AI, Shadow AI, physicians, qualitative

## Abstract

**Background:**

The rapid emergence of artificial intelligence (AI) has outpaced its formal adoption in health care organizations, contributing to the emergence of Shadow AI, defined here as the use of unauthorized AI tools by medical professionals. Under the European Union Medical Device Regulation, AI tools used for clinical purposes must undergo conformity assessment before use; general-purpose tools such as ChatGPT have not done so, rendering their clinical application unauthorized at the regulatory level. While Shadow AI offers potential efficiency gains and higher performance, it poses significant risks to data privacy, clinical safety, and regulatory compliance. Despite its growing prevalence, empirical research on the purposes for which physicians use Shadow AI remains scarce.

**Objective:**

This study explores the purposes for which physicians describe using Shadow AI in their work.

**Methods:**

We conducted a cross-sectional survey of physicians employed in Swedish health care organizations (N=357; response rate~64%). Data were collected between December 2023 and January 2024 via a verified online panel. We conducted a qualitative content analysis of free-text responses on the use of unauthorized AI tools. We applied theoretical lenses from the sociology of professions and paradox theory to interpret the empirical findings.

**Results:**

Physicians use Shadow AI for several purposes, which we grouped into 4 categories: clinical work and decision-making, administrative work, research and professional development, and technological interest and curiosity. More specifically, Shadow AI is used as a colleague and second opinion for clinical decision support (eg, differential diagnoses and rare cases), administrative tasks such as patient communication and documentation, and research aimed at staying up to date and exploring developments in generative AI. Physicians described using these tools compensated for perceived gaps in institutional systems, reducing workload, and accessing knowledge considered difficult to obtain through conventional channels. The findings reveal a tension between physicians’ drive to improve their practice and the regulatory and organizational constraints that render such use unauthorized.

**Conclusions:**

Shadow AI used by physicians presents both opportunities and risks for health care professionals and organizations. Shadow AI indicates gaps where formal hospital systems may fail to meet health care professionals’ needs and signals a way for physicians to strengthen their experience-based knowledge. It represents a renegotiation of professional boundaries, as physicians bypass institutional constraints to maintain professional efficacy. The findings highlight a paradox in which the same tools that pose regulatory and safety risks also address real gaps in clinical and administrative support, suggesting that governance approaches must account for this tension rather than relying on prohibition alone.

## Introduction

### Background

This paper addresses the use of Shadow AI by physicians, that is, the use of unauthorized artificial intelligence (AI) tools and systems. Recently, AI has become widely used in workplaces across numerous professions, organizations, and sectors [1]. In health care, AI technologies are increasingly integrated into physicians’ work, for example, improving diagnostic accuracy, reducing administrative burden by automating documentation tasks, and mediating patient interactions through virtual health assistants [2-4]. These applications demonstrate AI’s potential to improve physicians’ clinical capabilities and strengthen organizational performance, potentially making health care delivery more efficient and cost-effective [5]. However, the rapid proliferation of AI has raised concerns about the future of the medical profession and the risk of deskilling [6], particularly regarding the potential displacement of human clinical expertise and the pressing need to rethink medical education to balance AI literacy with the preservation of essential clinical competencies [7,8].

The emergence of large language models marked a turning point in the digital transformation of health care, as it introduced generative AI capable of producing human-like text, answering complex medical questions, and assisting with clinical documentation [9,10]. The rapid pace of innovation of new language models and applications has created a fundamental mismatch with health care organizations that operate within highly regulated environments characterized by slow, bureaucratic processes in technology adoption and institutionalization [11]. More precisely, many AI-enabled health care applications are legally classified as medical devices when they are intended for diagnosis, prevention, monitoring, prediction, or treatment. This brings them within the scope of medical technology regulation and subjects them to conformity assessment under the Medical Device Regulations (MDRs) in the European Union [12]. This regulatory-innovation tension has catalyzed the emergence of Shadow AI, a phenomenon that resembles Shadow IT, where employees use unauthorized technology solutions to meet operational needs in their daily work [13,14].

In health care settings, Shadow AI can be related to the deployment and use of AI tools and models by individuals or groups without formal institutional endorsement, governance oversight, or integration with existing clinical systems [15]. In the European Union, the unauthorized character of Shadow AI has a specific regulatory basis. Under the MDR, software intended for diagnostic, predictive, monitoring, or therapeutic purposes qualifies as a medical device and must undergo conformity assessment before clinical use (Regulation (EU) 2017/745, Art. 2(1)) [12]. General-purpose generative AI tools such as ChatGPT are not Conformité Européenne marked, have not undergone conformity assessment, and are not classified as medical devices. When physicians repurpose these tools for clinical reasoning or patient communication, they use applications that lack the legal basis for such use, rendering the practice unauthorized at the regulatory level, not merely informal or unendorsed at the organizational level. Furthermore, while the use of generative AI tools for personal development and administrative purposes is technically legal, digital tools are highly controlled through governance and institutional systems within the health care context. Shadow AI emerges when individuals use unauthorized AI tools to address immediate clinical challenges, streamline workflows, or improve productivity outside the explicit control of their organization or without conforming to the regulatory pathways required within the setting [16,17]. Consequently, the use of Shadow AI may not be monitored, evaluated, or even known by management and staff [18]. Shadow AI introduces unique risks related to AI’s capacity for autonomous decision-making, its potential to generate plausible but incorrect medical information, and its ability to process sensitive patient data through external systems [19]. This challenges traditional health care governance models that were designed for more predictable technologies [20].

The use of Shadow AI can be understood from a paradoxical perspective. Shadow AI promises to streamline clinical, communicative, and administrative work for physicians but may simultaneously introduce unforeseen, at times critical, risks. This positions Shadow AI as a “compromise” between sanctioned use and nonuse. However, limited research evidence and clinical experience, combined with the tendency for large language models to hallucinate, have led to delays in or abandonment of the adoption and use of AI by health care organizations. Consequently, the use of Shadow AI may lead to problematic outcomes in terms of, for example, increased security risks, ethical risks, and poor clinical decision-making [16]. At the same time, individual physicians may be interested in and motivated to investigate the potential benefits of AI-powered decision-making and AI-supported administration. Thus, there might be several reasons behind the adoption and use of Shadow AI in health care, and the concept embodies both risk and innovation.

Currently, our understanding of Shadow AI remains underdeveloped. Existing studies are predominantly conceptual [17,18] or draw on secondary data to examine legal implications from a sector-level perspective [19], leaving the phenomenon largely unexplored empirically. To the best of our knowledge, no scientific studies have directly explored the purposes for which physicians use Shadow AI in their daily practice, nor the motivations and concerns that accompany such use. This gap matters for 2 reasons. First, physicians are at the forefront of AI adoption [21], meaning that their informal use patterns may reveal both innovative applications and potential risks that formal organizational assessments fail to capture. Second, given that AI used for diagnostic or therapeutic purposes falls under the MDR, some Shadow AI practices may constitute regulatory violations, a dimension that conceptual work alone cannot adequately capture.

### Shadow AI Use by Physicians: Professional Work and Paradoxical Tensions

From a sociology of professions perspective, physicians represent a classic profession with clearly defined jurisdiction over diagnosis, inference, and treatment [22]. Central to professional work is discretion, that is, the freedom to make decisions based on specialized knowledge and professional judgment [23]. Discretion encompasses both a structural aspect (the ability to act within permitted boundaries) and a cognitive aspect (the ability to determine appropriate action through practical reasoning). As AI tools can alter routines and working methods [24], they potentially reshape the very foundation of professional work. Since agency is fundamental to professional work [23,25], the informal adoption of AI through Shadow AI practices raises critical questions about how physicians maintain professional discretion while leveraging AI capabilities. The sociology of professions lens is therefore useful, as it allows us to explore how Shadow AI adoption represents not merely a technological phenomenon, but a renegotiation of professional boundaries, autonomy, and the very nature of medical expertise.

Paradox theory highlights how organizations and professionals routinely confront tensions that are both contradictory and interdependent, and that cannot be fully resolved [26]. Classic paradoxical tensions include exploration versus exploitation, where the pursuit of novelty coexists with the need for reliability [27,28]; flexibility versus control, in which adaptive action must occur within structures that ensure coordination and accountability [29]; and change versus stability, reflecting how transformation depends on maintaining continuity in core practices [30]. These tensions persist because each pole enables and constrains the other, creating ongoing demands for balancing, shifting, or reframing. Paradox theory, therefore, offers a useful foundation for understanding settings such as health care, where professionals must engage emergent technologies while operating within systems that emphasize procedural rigor, professional responsibility, and institutional stability.

Without empirical data, Shadow AI is just a theoretical concept. Health care organizations need evidence to develop safety rules that encourage innovation while protecting patients, supporting health care professionals, and complying with regulations.

Against this backdrop, this paper aims to explore the purposes for which physicians describe using Shadow AI in their work.

## Methods

### Study Design and Setting

This study used a cross-sectional survey design targeting physicians currently employed in Swedish health care organizations. Sweden’s health care system is publicly financed and decentralized across 21 county councils, each responsible for health care provision and public health promotion. The system provides a well-organized institutional backdrop against which informal AI adoption practices can be examined [31].

The survey was conducted in collaboration with a certified polling company [32], which managed recruitment and administration through a closed, password-protected online platform. Potential participants were drawn from a verified online panel of 1890 health care professionals maintained by the polling company, who reached out to eligible physicians via email. Researchers had no direct contact with respondents and no access to their personal or contact information at any stage. Responses were captured automatically through the Confirmit Extranet Survey Designer platform and transferred to an anonymized dataset.

The questionnaire was developed iteratively. To assess item clarity, 2 online pilot interviews were conducted with practicing physicians (1 in dermatology and 1 in radiology) in July 2023 via Microsoft Teams. A soft launch of 50 questionnaires followed in November 2023 to test platform responsiveness and technical functionality. After confirming that all items, logic, and submission mechanisms functioned correctly, the full survey was launched in December 2023, with data collection continuing through January 2024.

The survey was organized into 6 sequential pages. Page 1 introduced the study, stated its purpose, identified the lead investigator, and assured participants that their responses would be fully anonymized and that no personal information would be collected or stored. Page 2 provided a structured overview of AI concepts and contemporary applications in clinical settings, ensuring that all respondents shared a common understanding of the technology before answering. Page 3 contained a single eligibility screening question confirming whether the respondent actively used AI in their work; those who did not were excluded from proceeding. Page 4 collected 4 demographic items: sex, age, clinical setting, and years of professional experience. Page 5 contained 2 open-ended questions designed to capture physicians’ experiences of how AI was currently used in practice and their expectations for AI support in clinical work: “How has AI changed the way you perform your work tasks?” and “What functions/tasks AI systems/tools perform that could make your work easier?” Page 6 contained 2 open-ended questions on barriers to AI adoption and motivations for its use in clinical settings: “What has mainly influenced your decision to use or not use AI?” and “What do you see as the main challenges to implementing AI in healthcare?”

These questions contextualized physicians’ informal AI use by focusing on the broader constraints and drivers that shape their engagement with AI. Respondents could navigate back through pages, and a summary review step was presented before final submission, allowing participants to check and amend their answers. Each participant received a one-time access code to prevent duplicate entries.

### Data Analysis

A conventional content analysis was used, in accordance with Hsieh and Shannon [33], to suit the data and the aim of the study. The method was chosen because of the relative novelty of the subject, with limited previous research, presenting a situation that called for an open approach to data. As knowledge from content analysis is grounded in data, its use aids in gaining and developing information from study participants without imposing a preconceived theoretical understanding [33]. To enhance the validity of the findings, investigator triangulation was used. A first round of coding revealed the unexpected finding that physicians admitted to using AI tools that were not formally procured or certified for use in a medical setting [32]. Thus, the coding revealed patterns of informal, unsanctioned AI use—referred to as Shadow AI. This led to a restart of the coding. First, the free text answers were read multiple times by all authors to achieve immersion in the material and to gain an overview of the full dataset. Codes were generated directly from the data, and no pre-established frameworks were applied. The authors met to discuss initial thoughts and conduct an initial analysis after the first coding. Further coding was done by 2 of the authors (LP and MH), after which meetings with all authors took place to discuss and formulate emerging and tentative codes. The authors together grouped and organized the data into categories.

The analysis was followed by theoretical interpretation in which the empirically derived categories were examined through paradox theory and sociology of professions. This followed the logic of conventional content analysis, where theoretical engagement occurs after categories have been established from the data [33]. The sociology of professions provided a lens for interpreting how Shadow AI use relates to professional jurisdiction, discretion, and autonomy, while paradox theory offered a framework for understanding the coexistence of contradictory demands that physicians navigate. Both frameworks are therefore mobilized in the discussion rather than embedded in the coding structure.

The categories were extensively discussed within the research team, with adjustments made until consensus was reached. Categories were chosen at the highest level of abstraction. To illustrate the findings, appropriate quotations from the data were chosen by the research team and translated into English by 2 of the authors, after which meetings took place to discuss and refine them and, in some cases, slightly amended to enhance comprehensibility. Amendments were limited to grammatical adjustments necessary for readability in English and were reviewed by all authors to ensure that the original meaning was preserved. The translation of the quotations was validated by a native English-speaking language editor. The research group was interdisciplinary and had experience in research in health care settings, information-driven care, and qualitative methods.

To clarify the basis for classifying the reported AI use, we note that the physicians’ descriptions of their use of AI were predominantly general-purpose generative AI applications, mainly ChatGPT, accessed through personal accounts and private devices. None of these tools were certified as medical devices, procured through organizational channels, or integrated into clinical IT infrastructure. Thus, they were classified as Shadow AI. In other words, the classification of their use as Shadow AI rests on 2 concurrent conditions: the absence of conformity assessment under MDR at the regulatory level and the absence of organizational procurement, endorsement, and governance oversight at the institutional level.

### Ethical Considerations

The study adheres to the Declaration of Helsinki [34] and the guidelines on research ethics issued by the Swedish Research Council [35]. Formal ethical review by the Swedish Ethical Review Authority (Etikprövningsmyndigheten) was not required under the Swedish Ethical Review Act (SFS 2003:460), as the study meets none of the 4 conditions that trigger mandatory review: it involves no physical intervention on living or deceased persons; it uses no method intended to affect participants physically or mentally, nor does it pose an obvious risk of harm; it involves no biological material traceable to any individual; and it does not process sensitive personal data as defined under General Data Protection Regulation Article 9—such as health data, ethnic origin, or political opinions—nor personal data relating to criminal offenses [36]. Participants were surveyed exclusively about their professional experiences and workplace practices. The study upholds the principles of informed consent and respect for privacy and was guided by the ethical principles of autonomy, beneficence, nonmaleficence, and justice [37]. Prior to participation, respondents received a detailed invitation from the polling company stating the purpose of the study, the identity of the lead investigator, the estimated completion time, the topics that would be covered, and a clear assurance that no personal identifying information would be collected or stored. Participation was entirely voluntary, and respondents were informed that they could withdraw at any time without providing a reason. All response data were managed exclusively by the polling company through its secure platform and stored in fully anonymized form; researchers received only anonymized outputs and had no access to any individual-level identifying information. Informed consent was obtained at the beginning of the questionnaire, upon participants confirming they had read the study information and agreed to proceed. A financial compensation of US $148.2 was offered to each participant who completed the questionnaire, consistent with standard panel practice. We used the CHERRIES (Checklist for Reporting Results of Internet E-Surveys) [38] to guide our survey reporting (Checklist 1).

## Results

### Study Participants

Participation was contingent on panel membership and voluntary response, constituting a purposive sample [39]. Eligibility was based on current AI use in clinical practice. Before proceeding, participants were presented with a brief overview of AI applications in health care, including intelligent diagnostic and triage systems, medical image recognition, risk stratification tools, smart wearable devices, and remote consultation support, to ensure a shared understanding of what constitutes AI use. Only physicians who confirmed using such technologies in their work were included in the sample.

A total of 557 physicians meeting the eligibility criteria were invited to participate, yielding 357 complete responses (response rate~64%). Only fully completed questionnaires were retained for analysis; responses where participants did not complete all pages were excluded. There was no time limit for completing the survey, and completion time was not recorded.

Respondents were predominantly male (221/357, 62%) and mid-career, with 55% (195/357) aged between 35 and 54 years. Professional experience varied widely, with approximately 54% (190/357) reporting 10 years of experience or fewer, 23% (79/357) between 11 and 20 years, and 21% (78/357) more than 21 years. Most respondents worked in public sector settings, with public hospitals representing the largest group (194/357, 54%), followed by public health centers (70/357, 20%) and private health centers (50/357, 14%). A smaller proportion were affiliated with academic or research institutions (24/357, 7%), private hospitals (12/357, 3%), or other settings (7/357, 2%). Table 1 provides an overview of the sample characteristics.

**Table 1.** Overview of the sample characteristics.

Demographic details	Values, n (%)
Sex	
Male	221 (62)
Female	136 (38)
Age (years)	
<24	1 (<1)
25‐34	64 (18)
35‐44	114 (32)
45‐54	81 (23)
55‐64	68 (19)
≥65	29 (8)
Main affiliation	
Public hospital	194 (54)
Private hospital	12 (3)
Public health center	70 (20)
Private health center	50 (14)
University or college or research institution	24 (7)
Other	7 (2)
Professional experience (years)	
<1	10 (3)
1-10	190 (54)
11-20	79 (23)
>21	78 (21)

Below, we present our findings in terms of experiences related to the use of Shadow AI in clinical work and decision-making, administrative work, research and professional development, and technological interest and curiosity.

### Clinical Work and Decision-Making

The use of Shadow AI in clinical work encompasses diagnostics, prediction, and second opinions. Physicians stated that they use Shadow AI as (1) a second opinion or a colleague to discuss differential diagnoses with, (2) to find and remember unusual or complicated cases and diagnoses, (3) for hard-to-diagnose symptoms, and (4) to improve diagnostics. Examples are included in Textbox 1.

Textbox 1. **Examples of how physicians describe that they use Shadow AI.**

“I entered de-identified data (medical history, examination [or observed findings/clinical findings], test results) into ChatGPT, which suggested various differential diagnoses” [Participant 58].

“I have used ChatGPT on occasion to get help with rare conditions in order to suggest possible diagnoses” [Participant 202].

“In some cases, if one is uncertain about a diagnosis, one can consult ChatGPT” [Participant 96].

“In connection with difficult-to-interpret symptoms, I have used ChatGPT (in English), which works better than in Swedish” [Participant 111].

These 4 aspects may overlap in practice, but reflect distinct orientations toward AI: as a conversational partner for reasoning, as a retrieval aid for rare knowledge, as a diagnostic check, and as a means of improving accuracy.

Expressed needs include risk prediction when intensive care is initiated and obtaining an overview of a patient’s medication.

*A useful feature would have been decision support for choosing treatment/medication in situations where many variables must be considered*.

[Participant 43]

Other demands for clinical work included the same aspects, especially support in relation to differential diagnoses and rare complicated diagnoses, and connecting to other data and tools. Longitudinal analysis of patients was expressed as a particular interest—an area that no one reported having undertaken so far.

### Administrative Work

In this category, the use of Shadow AI was related to communicative support and documentation. A large language model such as ChatGPT was used to make specialized medical language or terms more comprehensible to patients and their relatives.

*To generate letters for patients, information from complex radiology reports was entered into GPT, which summarized the information in patient letters in a way that is easier for them to understand*.

[Participant 103]

Shadow AI, such as ChatGPT, was used as a support when physicians needed to explain something.

*To help me explain to patients*.

[Participant 236]

Explicitly expressed needs included reminders for both physicians and patients regarding medication, certificates, annual visits, and tests. Optimizing the scheduling of visits and documentation in the electronic health record with AI support was also expressed as a need. No statements were made about whether they wanted or required an improved AI tool to be formally approved.

### Research and Professional Development

Shadow AI was used by physicians for research and professional development to update them on the state of the art in research and provide an overview of the latest research in their medical field. The findings may overlap with clinical work and decision-making, but focus on the physician’s general needs and interests, rather than specific cases. An example is:

*I used ChatGPT to find clinical knowledge and information, obtaining clearer, more distinct responses to support decision-making*.

[Participant 168]

Explicitly expressed needs include better support from AI for evidence-based interpretation across various domains, such as prediction, diagnostics, and treatment.

However, physicians noted that there was a risk that they may rely too heavily on AI, thereby forgetting their own clinical knowledge, and that younger colleagues may not develop their own experience-based knowledge at all, as they would be dependent on AI output.

*That younger colleagues do not develop clinical experience but, instead, rely on AI. Over time, there is a risk that a physician may be unable to make decisions without AI, and the balance will then be tipped*.

[Participant 141]

Physicians also expressed concerns about time and resources; for example, there is limited time to learn how and when to use AI, and the lack of (local) technical support.

### Technological Interest and Curiosity

Shadow AI was also used by physicians because of technological interest and curiosity. In several instances, they were driven by curiosity and wanted to explore new ways of working. This may overlap with the research and professional development category, but here the answers were not stated as a means to an end but, rather, as more of an intrinsic motivation. One finding was that several physicians had programmed their own tools to explore what kind of output they could get from AI.

*I have programmed tools myself, mostly via ChatGPT, but also other open-source AI tools. I use these tools to quickly obtain information before decision-making, when appropriate*.

[Participant 191]

Physicians also expressed curiosity about AI output and had tried the technology to assess its quality. AI was described as the future of medicine in terms of clinical decision-making, administration, and efficiency; thus, it was considered important to be at the forefront.

*AI can [/should] be seen as a compilation of the knowledge and experience of all clinicians. It will be implemented more in the future, and it is important to be involved from the start*.

[Participant 2]

However, there were also physicians who stated that it is important to evaluate the capacity of AI before it becomes officially implemented in health care. The 4 categories show that Shadow AI use extends from immediate clinical needs to longer-term professional development and personal exploration of AI.

## Discussion

### Principal Findings

This paper presents one of the first empirical studies exploring the purposes for which physicians use Shadow AI in health care practice. Building on empirical evidence from Swedish physicians, we advance the concept of Shadow AI by drawing on literature on Shadow IT, as well as emerging conceptual and opinion-based work on Shadow AI and generative AI in health care. Given the current lack of empirical research on Shadow AI in health care, our study contributes by grounding the phenomenon in physicians’ actual practices rather than hypothetical or speculative scenarios. To interpret these findings, we draw on paradox theory [26] and the sociology of professions [22]. Together, these perspectives help explain how physicians navigate tensions between innovation and compliance, autonomy and governance, and efficiency and accountability when using unauthorized AI tools in their work.

Our findings show that physicians use unauthorized generative AI tools across interconnected domains of professional activity: clinical work and decision-making, administrative work, research and professional development, and technological exploration, while technological curiosity functioned as a driver of adoption. Across these domains, Shadow AI is primarily used to compensate for the absence of formally approved, integrated, and accessible AI solutions within health care organizations. Rather than representing isolated or deviant behavior, Shadow AI emerged as a pragmatic response to organizational, technological, and regulatory gaps. The findings therefore suggest that Shadow AI is not peripheral to health care work but closely linked to physicians’ attempts to maintain efficiency, improve communication, strengthen clinical reasoning, and remain professionally updated in contexts where institutional systems are perceived as lagging behind technological developments.

To better understand this phenomenon, we compare Shadow AI with Shadow IT, which can be regarded as its organizational forerunner (Table 2). Similar to Shadow IT, Shadow AI emerges when frontline professionals perceive official systems as insufficient, inaccessible, overly bureaucratic, or poorly aligned with the realities of practice [16,19]. As shown in Table 1, both phenomena are driven by attempts to bypass slow procurement and governance processes, improve workflow efficiency, gain rapid access to information, and use technologies that are readily available and familiar to professionals. However, our findings also indicate important differences between Shadow IT and Shadow AI. Shadow IT largely involves static or compensatory digital tools, such as messaging platforms, cloud storage, or informal communication systems [40]. Shadow AI, by contrast, introduces generative and increasingly autonomous systems capable of producing reasoning, predictions, recommendations, and clinical suggestions [16,19].

**Table 2.** Overview of Shadow IT and Shadow AI^a^.

	Shadow IT	Shadow AI
Definition	The use of IT tools, devices, software, and services by health care professionals to complement corporate-provided IT resources without formal IT department approval or organizational endorsement [40].	The use of AI^b^ systems, tools, or models that are developed, accessed, or used without formal organizational authorization, governance, or regulatory oversight, often operating outside IT control structures and compliance frameworks [16,17,19]. *The use of AI-powered systems, tools, or models by health care professionals for clinical or administrative purposes in the absence of 2 concurrent conditions: conformity assessment under applicable medical device regulation at the regulatory level and organizational procurement, endorsement, or governance oversight at the institutional level*.
Why it is used	Bypass slow IT procurement processes [40] Dissatisfaction with official IT tools [41] Wide availability and familiarity with IT tools (eg, WhatsApp and Dropbox) [40] Access tools better suited to local or task-specific needs [42,43] Rapid access to patient information [42] Pressure from peers [44]	*Technological interest and curiosity (eg, to explore new ways of working*) To achieve rapid productivity gains in daily tasks [15,16] To bypass lengthy or restrictive AI approval processes [16] To experiment with cutting-edge technology [15,16] To compensate for lack of formal AI strategies [18,19] Wide availability and easy accessibility of generative AI tools [15]
Main applications	Communication and socialization with patients and other health care professionals [40,44] Retrieve, record, and store patient information [44] Personal cloud storage [40] Team management and coordination [44] Search for clinical and practical information sources [44,45]	*Clinical work and decision-making*: *diagnostics interpretation* [17-19] *to obtain second opinions* *to assess unusual or complex cases* to analyze patient data in general [19] triage [18] *Administrative work*: *communication support* *text generation and transcription* [19] *to optimize the scheduling of visits* task and workflow automation [15,16] *Research and professional development*: *to stay up to date on the state-of-the-art research in the medical field*

a The table was elaborated based on a combination of conceptual and opinion-based literature and the empirical findings of this study. Findings derived from our empirical material are presented in italics format, whereas findings from previous studies are accompanied by their corresponding references.

b AI: artificial intelligence.

This distinction is particularly important in health care because Shadow AI intersects directly with physicians’ professional jurisdiction over diagnosis, inference, and treatment [22]. Unlike unauthorized cloud storage or messaging systems, using a large language model such as ChatGPT for differential diagnosis or clinical reasoning directly affects domains in which accountability, evidence requirements, and professional responsibility are considerably higher [46]. From a sociology of professions perspective, Shadow AI represents not merely a technological workaround, but a renegotiation of professional discretion and expertise. Paradox theory helps explain why physicians engage with these tools despite the risks involved. Physicians face contradictory yet interdependent demands: they are expected to improve efficiency and quality of care through innovation while simultaneously adhering to governance systems and regulatory structures that evolve much more slowly than the technology itself. Shadow AI may therefore be interpreted as a situated response to the organizational misalignment between the pace of technological development and the pace of institutional adoption and approval.

Our findings further show that physicians use Shadow AI across several domains of professional practice. In clinical work and decision-making, physicians described using generative AI tools as a “colleague” or “second opinion” to support differential diagnoses, interpret difficult symptoms, analyze unusual cases, and improve clinical reasoning. These findings empirically support concerns raised in conceptual literature, suggesting that Shadow AI may increasingly influence clinical judgment and decision-making processes [17-19]. In administrative work, physicians used generative AI for communication support, patient-friendly explanations, text generation, and documentation assistance, consistent with previous discussions regarding workflow automation and reduced administrative burden associated with generative AI [16,19]. In research and professional development, physicians described using Shadow AI to remain updated on medical knowledge, synthesize information, and support evidence interpretation. Technological curiosity also emerged as an important driver, as some physicians actively experimented with AI tools, programmed their own applications, and explored AI capabilities beyond immediate clinical needs. Together, these findings suggest that physicians are not passive adopters of AI technologies, but active experimenters attempting to integrate generative AI into professional practice in ways that they perceive as meaningful and useful.

Within the European context, these findings carry important regulatory implications. Under the MDR [12], software used for diagnostic, predictive, monitoring, or therapeutic purposes qualifies as a medical device and requires conformity assessment before clinical use. None of the AI tools described by respondents were Conformité Européenne marked, formally procured, or subject to organizational oversight. Consequently, the unauthorized character of Shadow AI documented in this study is not solely a matter of organizational governance, but also structurally shaped by the regulatory framework itself. At the same time, our findings indicate that Shadow AI should not be understood as a homogeneous phenomenon. The different domains identified in this study represent varying degrees of regulatory exposure and organizational risk. Clinical decision-making activities fall most directly under MDR requirements and therefore represent the most problematic form of Shadow AI use, whereas administrative, research-oriented, and exploratory uses often occupy regulatory “gray zones” associated primarily with organizational governance rather than explicit legal prohibition. This differentiation is theoretically important because it demonstrates that Shadow AI exists on a spectrum ranging from relatively low-risk exploratory use to potentially high-risk clinical applications.

From this perspective, Shadow AI constitutes a professional governance problem rather than merely a technical one. Informal adoption at the individual level appears to be sustained by incentive structures, in which efficiency gains, professional benefits, and perceived improvements in patient care are immediate and visible, whereas accountability remains diffuse and organizational oversight limited. Importantly, the sociology of professions helps explain why prohibition alone is unlikely to eliminate Shadow AI use. Physicians’ professional discretion [23] and sense of jurisdiction over clinical practice [22] create strong normative grounds for acting in what they perceive as the patient’s best interest, even when doing so transgresses formal organizational boundaries. Shadow AI can therefore also be understood through the concept of unethical pro-organizational behavior [47,48], which involves individuals engaging in “bad” actions for good reasons. In using Shadow AI, physicians technically violate IT governance and data policies (unethical or noncompliant behavior). However, they often do so to improve patient communication, speed up administrative workflows, and enhance diagnostic accuracy (pro-organizational or pro-patient behavior). This example of professional autonomy [49] creates a complex governance paradox: the very behavior that puts the organization at regulatory risk is often driven by a desire to help the organization succeed in its mission of patient care.

A core contribution of this study is the finding that Shadow AI helps physicians navigate a persistent paradox: AI is widely perceived as beneficial; yet, formal access to validated and institutionally approved AI tools remains limited, slow, or procedurally difficult [50,51]. This creates conditions in which physicians informally use and experiment with AI technologies despite the absence of formal approval pathways. In practice, physicians may incorporate generative AI systems to strengthen their own knowledge, improve access to information, and support clinical reasoning processes [52]. In this sense, Shadow AI may contribute to the emergence of what could be described as an AI-supported “jurisdiction 2.0,” where algorithmic outputs increasingly shape professional judgment and decision-making [53,54]. Paradox theory helps explain this dynamic. Physicians face competing but interdependent demands that cannot easily be resolved by either ignoring the technology or fully embracing it without institutional safeguards. Shadow AI therefore becomes a practical “middle space,” allowing clinicians the autonomy [49] to explore AI’s potential without crossing formal thresholds that trigger compliance or institutional scrutiny, while still adhering to high professional and regulatory standards and maintaining professional discretion [23].

**Figure 1.**
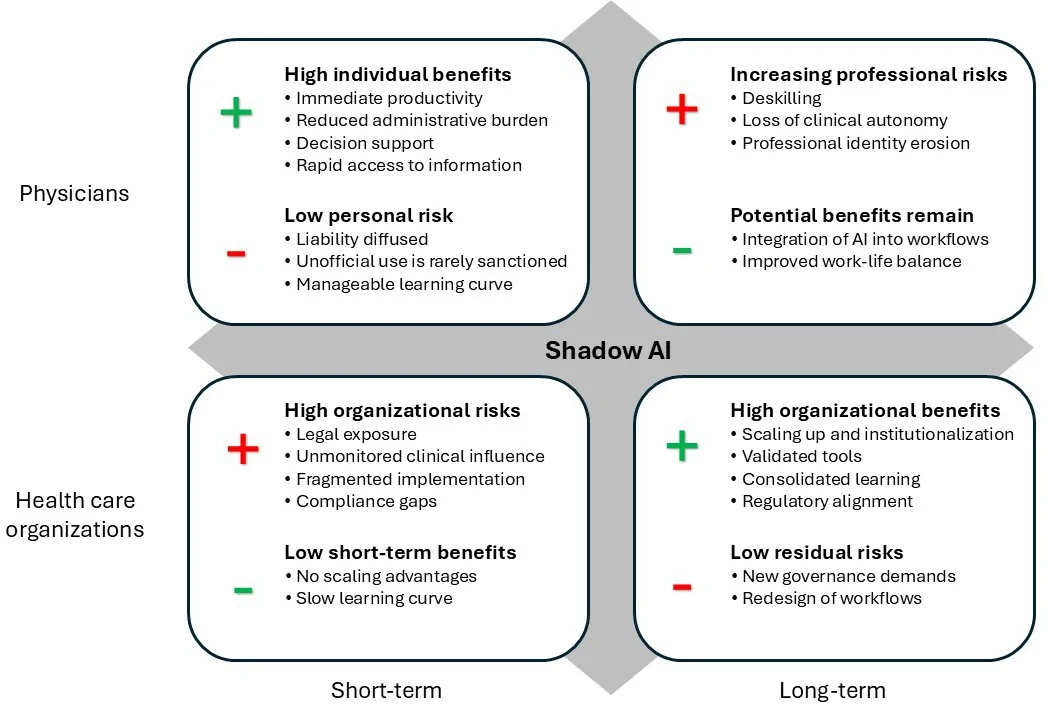
Inferred risk-benefit paradoxes of Shadow AI adoption in health care. AI: artificial intelligence.

Drawing on paradox theory and the sociology of professions, we further suggest that Shadow AI adoption may involve an uneven distribution of risks and benefits across professional and organizational levels and across time horizons (Figure 1). In the short term, physicians experience immediate and tangible benefits from Shadow AI use. While there are currently no empirical studies that specifically examine the benefits and risks of Shadow AI, existing literature on generative AI in health care indicates advantages that may also be applicable to Shadow AI adoption. These include a reduction in administrative burdens, enhanced cognitive support, improved productivity, faster access to information, and assistance with clinical reasoning [9,24,55,56]. Many of these tools may qualify as unauthorized for use without appropriate certification in clinical contexts under the MDR criteria used in this study. At the same time, personal accountability risks remain relatively diffuse and weakly enforced, particularly because organizational oversight of Shadow AI use is often limited or absent [18,19]. In contrast, organizations may disproportionately absorb risks that accumulate over time, including legal exposure, fragmented implementation practices, compliance gaps, cybersecurity vulnerabilities, and unmonitored AI influence on organizational workflows and decision support [17,57]. Conversely, in the long run, organizations may eventually benefit from physicians’ experimentation with and accumulated experience of AI tools. As a form of workaround practice, Shadow AI may function similarly to other persistent informal technology practices described in the workaround and Shadow IT literature, where unofficial experimentation can contribute to informal piloting, organizational learning, and double-loop learning processes that eventually support broader technology adoption and capability development [43]. Simultaneously, the literature on generative AI use in health care more broadly suggests that long-term risks may fall increasingly on physicians and the medical profession, manifesting as deskilling, dependency on AI outputs, erosion of professional autonomy, and shifts in professional identity and power perception [6,21,58]. The persistence of Shadow AI may therefore be explained by the asymmetrical distribution of benefits and risks: benefits are immediate and highly visible to physicians, whereas many organizational and professional risks remain delayed, diffuse, or difficult to observe in everyday practice.

Finally, an important implication of paradox theory is that health care organizations cannot assume that prohibiting unauthorized AI tools will eliminate their use. Restrictive governance approaches may instead intensify informal adoption when physicians perceive existing institutional systems as insufficiently responsive to practical clinical needs. In line with this, health care organizations may benefit from acknowledging the reality of Shadow AI and developing controlled and transparent environments for experimentation, such as clinical sandboxes, supervised testing pathways, or dedicated AI support structures. Clearer communication regarding permitted, recommended, and prohibited uses, together with investments in AI literacy and professional guidance, may support safer and more consistent practices [59,60]. Over time, understanding how physicians currently engage with Shadow AI may also help health care organizations identify unmet professional needs and inform the development of more relevant and clinically integrated AI systems. Addressing Shadow AI therefore requires governance approaches capable of engaging with the underlying paradox: the same conditions that make informal AI use professionally valuable are also the conditions that make it organizationally risky.

### Strength and Limitations

Our study has limitations that present opportunities for future research. First, while our qualitative analysis of 357 physicians’ responses provides valuable insights, the cross-sectional nature of our data collection offers a snapshot of AI adoption experiences at a single point in time. Additionally, there is a potential self-selection bias; physicians interested in technology are more likely to respond. However, the aim of this paper was not to generalize the results or count frequencies, but rather to present a deeper understanding of the phenomenon of Shadow AI, by conducting a qualitative analysis of free-text answers in a survey. Second, although our sample included physicians from diverse health care settings across Sweden, the study’s exclusive focus on a single national context may limit the transferability of our findings to other countries. Further research is needed to determine whether the results from this study are transferable to other countries. Longitudinal studies would be valuable to track how physicians’ perceptions, barriers, and drivers change as they gain more experience with AI systems over time. Third, the free-text format yielded responses that captured the purposes, motivations, and concerns associated with Shadow AI use, but rarely included detailed accounts of how physicians interact with AI tools in practice, for example, in terms of prompting strategies, iterative dialogue, verification routines, or integration into clinical workflows. This level of procedural detail would require interviews or observational methods designed to elicit accounts of situated practice. Future research adopting such approaches would complement the present findings by specifying the microlevel practices through which Shadow AI is enacted in clinical work. Fourth, AI is developing very rapidly. For example, while the use of ChatGPT has been stressed by respondents, other AI tools may become more relevant and used for Shadow AI activities. This implies that further research is warranted to follow the development of Shadow AI in parallel to formal AI use in health care.

### Conclusions

Physicians use Shadow AI in their work as a colleague and second opinion for clinical decision support, for administrative tasks, for professional development, and out of curiosity. Shadow AI use serves as an indicator of gaps where formal hospital systems do not fully meet the needs of the medical profession. While Shadow AI offers immediate benefits in reducing administrative burden and providing cognitive support, it simultaneously creates risks for physicians, the profession, and health care organizations. The 4 domains of use identified in this study carry different levels of regulatory exposure, ranging from clear regulatory violations in clinical application to organizational governance concerns in administrative and exploratory use. Theoretically, the study demonstrates that Shadow AI embodies a paradox of risk and innovation that cannot be resolved through prohibition alone, and that the sociology of professions helps explain how informal AI adoption represents a renegotiation of professional jurisdiction and discretion.

We argue that health care leaders should not view Shadow AI solely as a compliance violation but as a source of user-driven innovation that signals unmet professional needs. The path forward involves creating secure, monitored environments where physicians can experiment with AI tools safely, bridging the gap between the speed of AI development and the pace of medical governance. Governance approaches that engage with the underlying paradox, acknowledging that the same conditions that make informal AI use risky also make it professionally valuable, are more likely to succeed than blanket restrictions.

## Supplementary material

10.2196/93484 Checklist 1 CHERRIES checklist.

## Abbreviations

AI: artificial intelligence
CHERRIES: Checklist for Reporting Results of Internet E-Surveys
MDR: Medical Device Regulation
